# The osteoarthritis prevention study (TOPS) - A randomized controlled trial of diet and exercise to prevent Knee Osteoarthritis: Design and rationale

**DOI:** 10.1016/j.ocarto.2023.100418

**Published:** 2023-11-20

**Authors:** Stephen P. Messier, Leigh F. Callahan, Elena Losina, Shannon L. Mihalko, Ali Guermazi, Edward Ip, Gary D. Miller, Jeffrey N. Katz, Richard F. Loeser, Brian G. Pietrosimone, Sandra Soto, James L. Cook, Jovita J. Newman, Paul DeVita, Kurt P. Spindler, Jos Runhaar, Cortney Armitano-Lago, Vicky Duong, Faith Selzer, Ryan Hill, Monica Love, Daniel P. Beavers, Santiago Saldana, Aaron M. Stoker, Paige E. Rice, David J. Hunter

**Affiliations:** a J.B. Snow Biomechanics Laboratory, Department of Health and Exercise Science, Wake Forest University, Winston-Salem, NC, USA; b Thurston Arthritis Research Center, University of North Carolina at Chapel Hill, Chapel Hill, NC, USA; c Orthopedic and Arthritis Center for Outcomes Research, Department of Orthopedic Surgery, Brigham and Women's Hospital, Harvard Medical School, Boston, MA, USA; d Department of Health and Exercise Science, Wake Forest University, Winston-Salem, NC, USA; e Boston University School of Medicine, Boston, MA, USA; f Department of Biostatistical Sciences, Wake Forest School of Medicine, Winston-Salem, NC, USA; g Department of Orthopaedic Surgery, Thompson Laboratory for Regenerative Orthopaedics, Missouri Orthopaedic Institute, University of Missouri School of Medicine, Columbia, MO, USA; h Department of Kinesiology, East Carolina University, Greenville, NC, USA; i Clinical Research and Outcomes, Cleveland Clinic Florida, Weston, FL, USA; j Erasmus MC University Medical Center Rotterdam, Department of General Practice, Rotterdam, the Netherlands; k Sydney Musculoskeletal Health, Kolling Institute, University of Sydney, Sydney, Australia; l Rheumatology Department, Royal North Shore Hospital, Sydney, Australia

**Keywords:** Prevention, Osteoarthritis, Clinical trial, Women's health, Weight loss

## Abstract

**Background:**

Osteoarthritis (OA), the leading cause of disability among adults, has no cure and is associated with significant comorbidities. The premise of this randomized clinical trial is that, in a population at risk, a 48-month program of dietary weight loss and exercise will result in less incident structural knee OA compared to control.

**Methods/design:**

The Osteoarthritis Prevention Study (TOPS) is a Phase III, assessor-blinded, 48-month, parallel 2 arm, multicenter randomized clinical trial designed to reduce the incidence of structural knee OA. The study objective is to assess the effects of a dietary weight loss, exercise, and weight-loss maintenance program in preventing the development of structural knee OA in females at risk for the disease. TOPS will recruit 1230 ambulatory, community dwelling females with obesity (Body Mass Index (BMI) ​≥ ​30 ​kg/m^2^) and aged ≥50 years with no radiographic (Kellgren-Lawrence grade ≤1) and no magnetic resonance imaging (MRI) evidence of OA in the eligible knee, with no or infrequent knee pain. Incident structural knee OA (defined as tibiofemoral and/or patellofemoral OA on MRI) assessed at 48-months from intervention initiation using the MRI Osteoarthritis Knee Score (MOAKS) is the primary outcome. Secondary outcomes include knee pain, 6-min walk distance, health-related quality of life, knee joint loading during gait, inflammatory biomarkers, and self-efficacy. Cost effectiveness and budgetary impact analyses will determine the value and affordability of this intervention.

**Discussion:**

This study will assess the efficacy and cost effectiveness of a dietary weight loss, exercise, and weight-loss maintenance program designed to reduce incident knee OA.

**Trial registration:**

ClinicalTrials.gov Identifier: NCT05946044.

## 1 Backgrounds

Osteoarthritis (OA), the leading cause of disability among adults, has no cure and is associated with significant comorbidities [1]. Its increased prevalence and severity make it burdensome for people afflicted with the disease, and for health care organizations intended to administer care [2]. Obesity and OA were first linked in 1945 and this relationship has since been verified repeatedly [[3], [4], [5], [6]]. Obesity is a major risk factor associated with knee pain; people with obesity were 2.7 times more likely to have knee OA than adults without obesity [7].

**Fig. 1:**
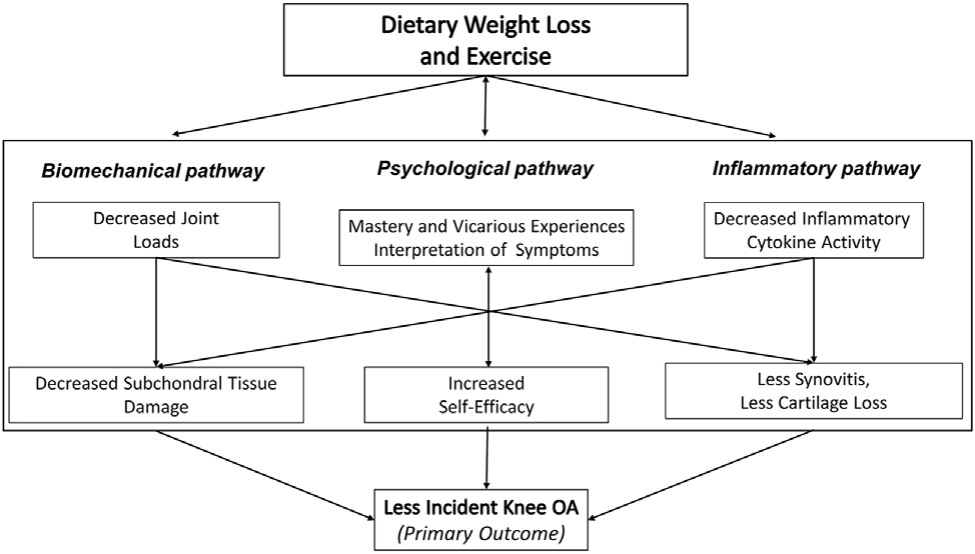
Mechanistic model by which dietary weight loss and exercise impact the biomechanical, psychological, and inflammatory pathways between weight loss and key clinical and structural outcomes.

Our mechanistic model, influenced by the seminal work of Griffin and Guilak [8], supports the premise that dietary weight loss and exercise may reduce knee joint loads, lower inflammation, and increase self-efficacy resulting in a lower incidence of structural knee OA (Fig. 1). Previous work showed that weight loss decreased knee joint loads such that every pound lost was associated with a 4-pound reduction in knee compressive forces while walking. The cumulative effect of this load reduction, over thousands of steps per day, reduces microdamage to the subchondral plate and calcified cartilage [9]. These areas are close to the overlying articular cartilage and likely protect articular cartilage integrity (Fig. 1).

In addition to the biomechanical pathway of larger joint loads with obesity, the modern concept of the role of obesity in chronic diseases, including OA, relates to a systemic low-grade pro-inflammatory state mediated by cytokines and adipokines produced by fat cells [10,11]. Even very low levels of these cytokines (pg/ml range) can negatively affect cartilage and bone metabolism. In our IDEA trial, we reported that intensive weight loss, with or without exercise, reduced knee joint loads, and systemic inflammation in patients with knee OA, thereby mitigating the inflammatory pathways involved in symptomatic OA [12].

Longitudinal observational data suggests that weight loss attenuates cartilage damage. A 5.1 ​kg weight loss over 10 years was associated with lowering the odds of developing symptomatic knee OA by 50 ​% [5]. Weight loss was associated with improvements in the quality (increased proteoglycan content) and quantity (reduced cartilage thickness losses) of medial articular cartilage in patients with obesity who underwent either surgically or non-surgically induced weight loss [13]. Gersing et al. [14] found that weight loss was associated with a reduction in degenerative cartilage changes 48 months and 96 months from baseline, with greater weight loss associated with less damage. Teichtahl et al. [15] noted that a 1 ​% weight loss over 2.3 years was associated with a reduced medial cartilage volume loss of 1.2 ​mm^3^ and improved knee symptoms while a 1 ​% weight gain was associated with increased medial cartilage volume loss of −1.2 ​mm^3^ and worsened knee symptoms. These results suggest that in people with obesity, even relatively small amounts of weight loss can potentially portend disease-modifying effects on knee joint structure and symptoms.

The addition of a psychological pathway to our mechanistic model recognizes the role played by self-efficacy in the development and progression of chronic diseases such as knee OA. Diet and exercise interventions designed within a social cognitive framework have shown that an intervention of diet-induced weight loss combined with exercise (D ​+ ​E) was significantly better for increasing self-efficacy than either exercise (E) or diet-induced weight loss alone (D). The treatment effects of D ​+ ​E on clinical outcomes were mediated by changes in self-efficacy over the course of the trial [16]. These collective findings along the biomechanical, inflammatory, and psychological pathways suggest that a combined intervention of diet-induced weight loss and exercise will provide a crucial strategy to reduce incident knee OA in at-risk females.

We address knee OA disease prevention in adult females because prevention of OA is preferable to treatment [17], females are affected at nearly twice the rate as males [18], and to date interventions designed to slow or stop knee OA progression have failed [[19], [20], [21]]. The objective of this multi-center randomized clinical trial is to establish the efficacy of a dietary weight loss, exercise, and weight-loss maintenance program designed to reduce the incidence of structural knee OA (defined as tibiofemoral and/or patellofemoral OA on MRI). Forty-eight month structural, symptomatic, and mechanistic outcomes will be compared between the intervention group and a control group; a cost effectiveness analysis will assess the value of the intervention from societal and payor perspectives.

## 2 Methods/design

### 2.1 Trial organization

The intervention is delivered in four centers in the United States and Australia. Clinical and Data Coordinating Centers oversee the day-to-day operation of the trial. The Executive Committee [Principal Investigator (PI), site PIs] is responsible for major policy and budgetary decisions that govern the conduct of the trial. An advisory board composed of females at risk for knee OA, health providers, public and private arthritis organizations, and industry ensures strong input from a variety of stakeholders.

## 3 Research design and methods

### 3.1 Trial design

The Osteoarthritis Prevention Study (TOPS) is a Phase III, assessor-blinded, multicenter randomized clinical trial with two parallel arms designed to establish the efficacy of a dietary weight loss, exercise, and weight-loss maintenance intervention in the prevention of structural knee OA in females at risk for the disease compared to a control group. Trial oversight is provided by a data and safety monitoring board (DSMB) appointed by the primary study sponsor (National Institute of Arthritis and Musculoskeletal and Skin Diseases). The institutional review board of Wake Forest Health Sciences approved this protocol (No. 80136).

### 3.2 Study sample

Participants include 1230 ambulatory, community-dwelling females with obesity (BMI ≥30 ​kg/m^2^), and aged ≥50 years. Structural and symptomatic eligibility is determined at the individual knee level. An eligible knee will have no radiographic and no knee (tibiofemoral and patellofemoral compartments) OA as detected by MRI [19] with no or infrequent knee pain (<15 days/month) in the same knee. Exclusion criteria are noted in Table 1.

**Table 1:** Exclusion criteria.

Exclusion	Method
• Symptomatic or severe coronary artery disease • unable to walk without a device • blindness • type 1 diabetes • active treatment for cancer • during the past 12 months knee fracture, ACL, MCL, or meniscus injury with or without surgical repair • knee injection during the past 6 months • bilateral knee OA by x-ray KL ​≥ ​2 • bilateral knee OA by MRI • bilateral symptomatic knee OA (frequent bilateral knee pain >15 days per month) • BMI< 30.0 ​kg/m2 • male sex • claustrophobia • contraindication to MRI • including body weight >300 lbs. • MRI knee coil does not fit	• Medical history • Posteroanterior fixed flexion knee and skyline view x-rays • MRI using MRI OA definitions, fit knee coil for MRI
• Unwillingness or inability to change eating and physical activity habits due to environment • cannot speak and read English	• Questionnaire, • assessment by interventionists
• Planning to leave area >2 months during the 48-month intervention period	• Questionnaire
• Current or recent weight loss intervention (weight loss surgery previous 6 months; weight loss program or weight loss medication previous 3 months)	• Questionnaire

### 3.3 Randomization and masking procedures

Each eligible participant is randomized with a 1:1 allocation ratio to either a dietary weight loss, exercise, and weight-loss maintenance intervention or a control group using a permuted block randomization approach with random block sizes and stratified by intervention site to ensure balanced allocations within sites and obesity categories. A web-based randomization system will determine group assignment. Outcome assessors will remain blinded throughout the trial with no access to participants during intervention visits.

### 3.4 Intervention centers

Clinical intervention centers include Brigham and Women's Hospital, Boston, MA; the University of North Carolina at Chapel Hill, Chapel Hill, NC; the University of Sydney, Sydney, Australia; and Wake Forest University, Winston-Salem, NC. Each site is metropolitan and surrounded by non-metropolitan rural and small city areas.

### 3.5 Clinical and Data Coordinating Centers

The Clinical Coordinating Center is located at Wake Forest University in Winston-Salem, NC and is responsible for overseeing the day-to-day operation of the trial including recruitment, randomization, adherence, retention, organizing training sessions, conducting intervention monitoring visits to ensure fidelity, and coordinating intervention resources.

**Fig. 2:**
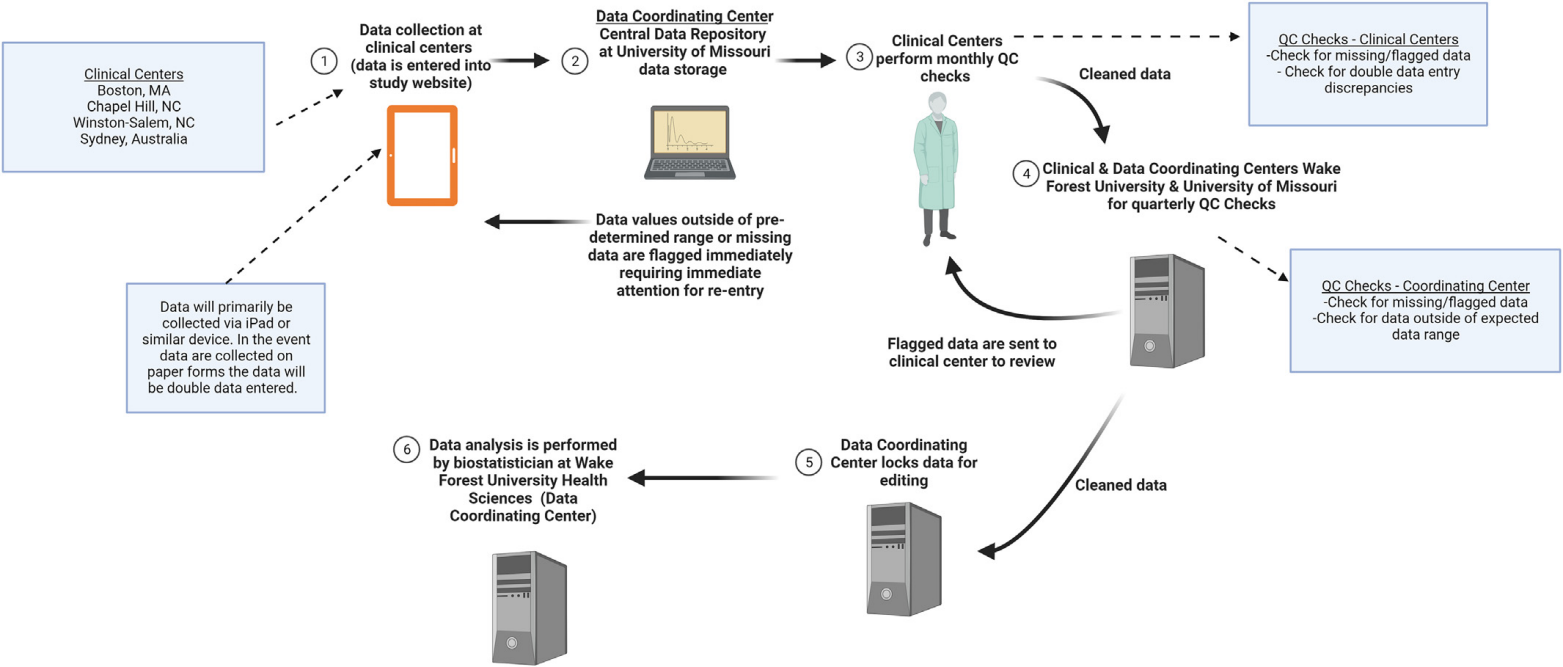
Flow of data from initial collection to final analysis. Created with BioRender.com.

The Data Coordinating Center is located in Columbia, MO at the University of Missouri Orthopaedic Institute and in Winston-Salem, NC at Wake Forest University Health Sciences. The Data Coordinating Center is responsible for developing a data system, storing data at the Missouri location from the four clinical centers, monitoring timely data collection, and ensuring efficient, secure, and reliable data entry and management. The biostatistical team is part of the Data Coordinating Center and is located at Wake Forest University Health Sciences. The biostatistical team, composed of Ph.D. and master's level biostatisticians, has full access to the data stored at the Missouri location. The biostatistical team is responsible for creating reports for the study team and the DSMB and developing and implementing the statistical analysis plan. Radiographic and MRI images are acquired at each study center, and then stored electronically at the Wake Forest University Health Sciences Imaging Core. The study radiologist accesses these images to determine eligibility at baseline and OA incidence at 48-month follow-up (Fig. 2).

### 3.6 Interventions

The intervention groups include diet and exercise (D ​+ ​E) and control (C). The weight-loss goal for the diet and exercise group is a minimum of 10 ​% of baseline body weight by the end of year 1 as recommended by the National Institutes of Health for adults with overweight and obesity [22]. The weight-loss goal is also consistent with our previous results in which a mean 11.4 ​% weight loss reduced knee pain by 51 ​% in patients with knee OA [12]. The weight-loss phase is followed by 3 years of weight-loss maintenance, with the goal of sustaining the achieved weight loss. The C group is modeled after our previous studies’ control groups, providing attention, social interaction, and healthy lifestyle classes [23,24].

#### 3.6.1 Dietary weight loss and exercise

Our previous trials helped establish diet plus exercise as part of standard of care for people with knee OA and obesity [25]. The dietary weight loss component of the intervention is characterized by the frequency of contacts, methods to induce dietary restriction, and behavioral therapy strategies. The first 9 months of the D ​+ ​E program is an energy-restricted diet with the option of using partial meal replacements and nutritious snacks (Rapid Nutrition, PLC). This individualized program is based on similar diet programs used in our previous trials in which most participants reached their weight loss goal between 9 and 12 months after baseline [12,26]. After reaching their weight loss goal, participants may either begin weight-loss maintenance, or may continue to lose additional weight using safe and healthy nutrition practices. To mitigate the possible health risks of rapid and extensive weight loss (e.g., detrimental loss of muscle and bone mass) [27], established alert values include an average weight loss greater than 4 ​kg/week; weight loss >20 ​% of body weight at 6-month follow-up; and a weight loss >30 ​% at any point during the study. The initial diet plan includes an energy-intake deficit of 500 ​kcals/day from an estimated energy expenditure (predicted resting metabolism x 1.2 activity factor) and the lowest intake is set at 1100 ​kcals/day. The kcal distribution is 15–20 ​% from protein, with at least 1.2 ​g protein/kg of ideal body weight; <30 ​% from fat; <10 ​% from saturated fatty acids; and 45–60 ​% from carbohydrates. These levels are consistent with the Dietary Reference Intakes for Energy and Macronutrients and successful weight-loss programs [28,29]. The exercise program is designed for participants to expend an average of 100 ​kcals/day, for a total deficit of at least 600 ​kcals/day.

Group and individual sessions with the intervention staff are conducted throughout the 48 months. Initially, content emphasizes nutrition and behavioral strategies to attain weight-loss goals and is followed by behavioral strategies for weight-loss maintenance. Topics for these maintenance sessions include how to elicit social support, problem solving, overcoming barriers, self-accountability, and ways to build self-efficacy for maintaining weight-loss over time. The number of optional meal replacements and the number of contacts are noted in Table 2.

**Table 2:** Summary of diet-induced weight loss plan and number of planned contacts.

Months	Weight loss plan	Meal Replacements per day (N)	Contacts per month (N)		
			Total	Individual	Group
0–9	Energy restriction 500–600 ​kcals/day	1–2 (optional)	4	1	3
10–24	Either continued energy restriction or weight loss maintenance once 10 ​% weight loss reached	1 (optional)	2–3^a^	1	2
25–36	Energy restriction/weight-loss maintenance	0–1	1–2^a^	1	1
37–48	Weight-loss maintenance	0–1	1^b^	1	1

a Individual sessions transition to every other month once 10 ​% weight loss is reached.

b Group and individual sessions will alternate every other month.

The exercise component includes two 60-min sessions per week for 48 months. For the first 24 months, participants exercise 2 days/week supervised by study interventionists at one of the designated facilities. For months 25–48, participants wishing to transition to exercise at another location (e.g., home or community gym) will alternate between the supervised facility and a location of their choice during a 2-month transition phase. Previous experience indicates that most participants will choose to maintain the combination of the supervised facility with an alternate location, with about 10 ​% opting for an alternate location entirely [12,26]. The exercise program prescribed to each participant consists of aerobic (15 ​min), resistance-training (20 ​min), a second aerobic (15 ​min), and cool-down (10 ​min) phases. Participants are encouraged to exercise on their own most other days of the week.

Interventionists, trained in validated behavioral change techniques based on a social cognitive theory framework [30], will work with participants in the D ​+ ​E group to gain confidence in their ability to maintain weight-loss for the long-term, maintain exercise independently, and establish a routine of healthy diet behaviors.

#### 3.6.2 Control group

The C group (presented to participants as the Healthy Living Group) is modeled after our previous control groups [23,24,26] and provides attention, social interaction, and lifestyle classes. There will be four, 60-min face-to-face group meetings per year, and quarterly newsletters and text messages to keep the participants engaged. These interactive sessions and the newsletters convey information on topics ranging from medication management, health screenings, menopause, sleep hygiene, to chronic disease management.

### 3.7 Adherence and retention

A social cognitive conceptual framework is used as the foundation for implementing problem-solving strategies and structuring a positive environment for the D ​+ ​E and C groups [30,31]. Interventionists receive training and ongoing supervision from the study health psychologist in validated behavioral techniques to enhance adherence to the D ​+ ​E intervention, and the C group sessions. Adherence is defined as attendance to sessions divided by the number scheduled; for the D ​+ ​E intervention it is defined as attendance to the diet classes and exercise sessions; for the C group it is attendance to the lifestyle group sessions. Make-up sessions are available to participants in both groups when there are scheduling conflicts.

Retention in the study is guided by social cognitive theory concepts with the use of incentives and building valued expectations for participation throughout the 48 months. Study staff strive to schedule testing appointments to accommodate the schedules of each participant, with reminder calls as necessary. When participants cannot attend a full testing session, abbreviated testing is offered in a phased approach to maximize data collection and retention. Retention is defined as the number of participants that complete at least one 48- month follow-up measure divided by the number of participants randomized x 100.

### 3.8 Intervention fidelity

Clinical Coordinating Center personnel provide on-site and virtual training for the interventionists at the US centers on the delivery of the dietary weight loss, exercise, and weight-loss maintenance interventions and the control group sessions [32,33]. For the Sydney center, training occurs via Zoom meetings and is augmented with an in-person site visit performed by the senior project manager during the first year. Interventionist are trained and tested on the contents of a detailed treatment manual that includes procedures for screening, data collection, intervention delivery, safety, and good clinical practice. Once interventions begin, interventionists will complete a checklist for each session; record the number of sessions and their length; and report any deviations from the planned protocol. Training will also include examining principles and techniques of behavior change, methods to facilitate group interaction, and strategies for working with participants with different needs. Interventionists meet monthly with the study health psychologist via Zoom to discuss their experiences and facilitate consistency among sessions and centers. Intervention sessions are videotaped quarterly and reviewed for consistency.

### 3.9 Trial conduct

#### 3.9.1 Recruitment

The recruitment goal for each center was based on previous recruitment successes; Chapel Hill, Winston-Salem, Boston, and Sydney preliminary recruitment goals are 300, 360, 250, and 320, respectively (total ​= ​1230). Based on our mock recruitment and previous recruitment experiences, we estimate an average yield (number randomized divided by the number who expressed interest in the study) of 13 ​% (Table 3). A web-based data tracking system will monitor recruitment strategies at each center.

**Table 3:** Preliminary recruitment projections.

Inquiries	Eligible after prescreening	Eligible after Screening Visit 1 including X-ray	Eligible after Screening Visit 2 including MRI	# Randomized
9110	2733 (30 ​% of inquiries)	1640 (60 ​% of pre-screened)	1230 (75 ​% of SV1)	1230 (13 ​% of inquiries)

Inquiries is defined as the number of people who expressed interest in the study.

The four center project managers and the coordinating center staff will coordinate recruitment efforts at each clinical center. Recruitment methods include mailings, local newspaper ads, hospital patient databases, and social media platforms. We also have strong partnerships with worksites in each community, access to churches, and a large database of adults aged ≥50 years who have signed consent to be contacted about participating in future clinical trials at each center. The Clinical Coordinating Center will build and maintain a study website and web-based tracking system where data at each center are entered in real-time for review of adherence to recruitment goals, intervention adherence, and study retention.

New participants are enrolled in the trial in waves approximately every 3 months during the recruitment period. The use of waves, as opposed to starting participants in their respective interventions as soon as they are randomized, promotes a group dynamic that is designed to enhance adherence to the intervention and retention in the trial [31]. Overall, the goal is to enroll an aggregate of 246 participants per wave for five waves.

### 3.10 Measurements

#### 3.10.1 Screening radiograph and MRI

To be eligible for participation, subjects must have at least one knee free of radiographic and MRI evidence of knee OA. Bilateral posteroanterior (PA) weight-bearing knee x-rays using a positioning device and the modified Lyon-Schuss technique [34] will be used to identify tibiofemoral (TF) OA and skyline views to identify patellofemoral (PF) OA. We will exclude potential participants with bilateral PF OA [Joint Space Narrowing (JSN) ​≥ ​2 on OARSI scale, definite osteophytes on the lateral and/or medial patella [35]] or TF OA (Kellgren-Lawrence score (KL) ​≥ ​2) [36]. For two eligible knees, a hierarchal approach using the TF score (KL 1, 0), PF score (1, 0), and pain (most symptomatic) will determine the index knee for MRI analysis. The dominant leg will be selected if x-ray and pain are equal. For x-rays that indicate no or doubtful knee OA (KL ​≤ ​1), the absence or presence of MRI-defined OA is determined using the Rapid OsteoArthritis MRI Eligibility Score (ROAMES) semi-quantitative scoring system (note: the ROAMES is used to assess eligibility only) [37]. If the most at-risk knee is not eligible after the MRI, the other knee may be reassessed. Based on the Prevention of Knee Osteoarthritis in Overweight Females (PROOF) trial, 20 ​% of those who pass the x-ray will present with MRI-based knee OA and be ineligible for randomization [38,39].

#### 3.10.2 Primary outcome

The primary outcome is incident structural knee OA assessed using the MRI OA definition (Table 4). The MRI Osteoarthritis Knee Score (MOAKS) is used to determine the severity of individual OA features on MRI. Using these scores, the presence of OA on MRI is determined using the definition presented in Table 4 [19]. Incident disease is defined as the development of tibiofemoral and/or patellofemoral OA on MRI.

**Table 4:** The MOAKS scoring system to determine MRI Defined Knee Osteoarthritis [17].

Tibiofemoral (TF) compartment	Patellofemoral (PF) compartment
The presence of both group [A] features or one group [A] feature and ≥2 group [B] features in the TF compartment: Group A 1 A definite osteophyte^a^ 2 Full thickness cartilage loss^b^ Group B 1 Sub-chondral bone marrow lesion not associated with meniscal or ligamentous attachment^c^ 2 Meniscal subluxation, maceration or degenerative tear^d^ 3 Partial thickness cartilage loss (where full-thickness loss was not present)^e^ 4 Bone attrition	All of the following in the patella or anterior femur: i) A definite osteophyte^f^ ii) Partial or full thickness cartilage loss^g^


a Osteophyte size grade ≥2 in ≥1 ​TF-subregion.

b Full-thickness cartilage loss grade ≥1 in ≥1 ​TF-subregion.

c Bone marrow lesion size ≥1 in ≥1 ​TF-subregion.

d Meniscal extrusion grade ≥1 and/or presence of any meniscal pathology.

e Cartilage loss size grade ≥1, with no full-thickness cartilage loss, in ≥1 ​TF-subregion.

f Osteophyte size grade ≥2 in ≥1 ​PF-subregion.

g Cartilage loss size grade ≥1 in ≥1 ​PF-subregion.

Radiographic features such as loss of joint space represent relatively late anatomical changes associated with OA and are preceded and detected with greater sensitivity with MRI [[40], [41], [42], [43]]. Unlike x-rays, MRI provides a direct 3D assessment of knee cartilage loss as well as visualizing other non-cartilaginous joint changes including bone marrow lesions, osteophytes, synovitis, and meniscal pathology that are more closely associated with symptoms and disease progression [39]. MRI is a widely used measure of structural joint changes as it has proved to be a valid and reproducible technique for knee OA [44]. A definition of MRI-defined OA was proposed to facilitate earlier detection, prior to radiographic disease [19]; by combining semi-quantitative scores of important OA features (e.g. osteophytes, cartilage loss, bone marrow lesions) the MRI-based definition of OA can be used to define the presence/absence of OA in the tibiofemoral and patellofemoral compartments (Table 4).

Recent population-based studies show that a proportion of radiographically normal knees have osteophytes and cartilage damage detectable by MRI. That is, MRI is more sensitive than radiographs, and has better content validity, in that it includes both soft and hard tissue measures that define OA more completely [45]. These structural changes are important because many cannot be directly visualized on radiographs and these MRI methods have been advocated in recent early OA definitions by OARSI [46]. Data from the PROOF trial showed overall incidence of clinical and radiographic knee OA of 12 ​% and 19 ​% among middle-aged women with overweight/obesity, respectively. In knees with incident OA after 30 months, using the MRI-based OA definition that we will use in TOPS (Table 4), the incidence of clinical and of radiographic knee OA was 22 ​% and 30 ​% after 80 months, respectively. This shows that our MRI outcome is predictive of future incidence of ‘established knee OA’ using validated outcomes [38].

All radiographs and MRIs are uploaded to a central repository located at the Wake Forest Imaging Center for readings by the study radiologist. MRIs are performed on either a 1.5 or 3 ​T Scanner and are analyzed using the MOAKS semiquantitative scoring system (Table 4) [47].

#### 3.10.3 Secondary outcomes

Prespecified secondary outcomes include the assessment of knee OA symptoms using The Knee Injury and Osteoarthritis Outcome Score (KOOS) knee function subscales including KOOS pain [48], 6-min walk distance, and health-related quality of life measured with the SF-36 questionnaire [49]. Plasma levels of IL-6 by ELISA - a measure of inflammation [12,23], knee compressive force - a measure of knee joint loading measured using gait analysis and musculoskeletal modeling [12,24,50], and self-efficacy for weight loss and exercise [16] are mechanistic secondary outcomes (Table 5). A cost-effectiveness analysis [51] will establish the value of the 48-month D ​+ ​E (diet and exercise) intervention.

**Table 5:** Measurements with screening (0 months) and follow-up visits (12, 24, 36, 48 months). Logistics and testing fidelity procedures are located in the Data Management Plan.

Measurements	Explanation	Testing (month)
**Primary Outcome**		
MRI	See Table 4 for details	0, 48
**Secondary Outcomes**		
KOOS pain	The Knee Injury and Osteoarthritis Outcome Score clinical knee pain score [74,75]	0, 12, 24, 36, 48
6-min walk	Measure of mobility	0, 12, 24, 36, 48
Blood biomarkers^a^^,^^b^	Measure of inflammation (IL-6) [76]	0, 48
Gait testing^a^^,^^c^	Measure of knee joint loading [46,77,78]	0, 48
SF-36	Short Form Survey, Health-related quality of life [45]	0, 12, 24, 36, 48
Weight loss self-efficacy, Exercise self-efficacy	Self-efficacy for diet and weight loss (79) and exercise (80)	0, 12, 24, 36, 48
**Questionnaires for Cost Effectiveness Analysis**		
Cost Effectiveness	See Economic Evaluation section for details	0, 12, 24, 36, 48
EuroQol Quality of Life(EQ5D)	Quality of life measure (81) required for the cost effectiveness analysis	0, 12, 24, 36, 48
Work History Resource	Visits to clinicians, tests, medications, injections, surgery, alternative therapies (82)	0, 12, 24, 36, 48
Work Productivity and Activity Impairment Index	To assess absenteeism and presenteeism (82)	0, 12, 24, 36, 48
**Additional Questionnaires**		
Informed Consent		0
Eligibility Questionnaire	To determine eligibility	0
Demographics		0
Medical, Weight History	For eligibility and to document changes in health including frequency of knee pain	0, 12, 24, 36, 48
Medication form	Atherosclerosis Risk in Communities (83)	0, 12, 24, 36, 48
PASE scale	Physical Activity Scale for Elderly (84,85)	0, 12, 24, 36, 48
SWL	Satisfaction with life scale (86)	0, 12, 24, 36, 48
PSS	Perceived stress scale (87)	0, 12, 24, 36, 48
PANAS	Positive and Negative Affect Scale (88)	0, 12, 24, 36, 48
KOOS subscales	KOOS subscales including symptoms, ADL, Sport/Rec [44,74]	0, 12, 24, 36, 48
Safety from Crime	Neighborhood safety (89)	0, 12, 24, 36, 48
Walkability and Exercise Environment Scale	Neighborhood walkability (89)	0, 12, 24, 36, 48
Social Cohesion Scale	Neighborhood social cohesion (90)	0, 12, 24, 36, 48
Dietary screener	Dietary screener from NHANES 2009–2010 (91)	0, 12, 48
Health Literacy	Functional health literacy measure (92)	0
CES-D	Center for Epidemiological Studies Depression (93)	0, 24, 48
Adverse events	Collected as they occur	
**Additional Tests/Imaging**		
Height	To determine BMI	0
Weight	To determine BMI	0, 12, 24, 36, 48
Urine biomarkers^c^	Stored for subsequent analysis	0, 48
Knee PA, Sunrise x-rays	Screen for initial eligibility to undergo MRI exam	0
DXA^d^	To measure changes in body composition and BMD	0, 24, 48

a Methodology for knee joint loading and IL-6 measures can be found in our previous studies [12,20].

b Measured at the 3 US sites (N ​= ​910).

c Measured at the Winston-Salem and Chapel Hill sites (N ​= ​660).

d Measured at the Winston-Salem site (N ​= ​360).

To reduce biomechanical gait variability, a designated tester at each gait testing site (Wake Forest and UNC sites) will assume responsibility for the collection of gait data. To ensure the precision of marker placement and reliability between both sites, test-retest comparisons will be conducted during the 3-month planning period utilizing the same participant with obesity. Furthermore, the Bell regression equation set will be employed to facilitate a more accurate estimation of hip joint centers in participants with obesity [52,53]. All data analyses will be centralized at the Wake Forest location.

Additional measurements that are not included in the specific aims are height and weight, body composition, and questionnaires to assess eligibility, demographics, psychosocial outcomes, social determinants of health, dietary intake, additional KOOS subscales, and adverse events (Table 5).

### 3.11 Economic evaluation

A validated computer-simulated model of incident knee OA will establish the cost-effectiveness of this intervention using data from the TOPS trial. The cost-effectiveness ratio provides a measure of value. The cost-effectiveness of a specific prevention or treatment strategy is measured in dollars per quality-adjusted life-year gained ($/QALY). The choice between policy alternatives is best made via incremental analysis, defined as the difference in direct medical costs between two strategies divided by the difference in quality-adjusted life expectancy. To determine how OA prevention-related expenditures compare with other uses of resources, the incremental cost-effectiveness ratios are compared with those in other areas of health care.

If the D ​+ ​E intervention is found to be effective, a budget impact analysis (BIA) of the D ​+ ​E program will quantify the financial consequences of adopting this program by various payer models, including insurance organizations, health care systems, and government. BIA will predict how adoption of the D ​+ ​E intervention will impact the trajectory of spending for people at a high risk for knee OA. These results could be used for budget planning and changes in health insurance premiums. The OAPol model will also quantify the extended financial benefits of weight loss reduction beyond reduction in pain and improvement in functional status, including the reduction in incident cardiovascular disease and type 2 diabetes.

Data are collected from all 4 clinical centers on resource utilization over 6-month intervals for the duration of the intervention and 2 years post-intervention. These data are used as input parameters for a ‘country’ specific analysis using the data from the US to inform US-centered cost-effectiveness analysis, and data from Sydney to inform Australia-centered cost-effectiveness. Direct medical costs include costs of inpatient stays and procedures, emergency department visits, outpatient physician visits as well as costs of laboratory studies, medical devices, and prescription and non-prescription medications. Intervention costs include the cost of meal replacements, progress monitoring, and wages of personnel delivering weight loss, exercise, and weight-loss maintenance interventions over the 4-year intervention period. The cost also includes any facility rental costs required to deliver the intervention.

Direct non-medical costs and indirect costs include the cost of transportation to intervention centers and costs related to the time that study participants need to engage in all TOPS activities. Participant data on lost wages and productivity losses for those working that are related to knee pain are also included in this category.

Utility measures capture patients’ preferences for health states and are scored from 0.00 (death) to 1.00 (perfect health). Utility will be derived from EuroQol (EQ-5D) [54]. The EQ-5D comprises 5 dimensions (mobility, self-care, usual activities, pain/discomfort, anxiety/depression). Health states defined by the 5-dimensional system are converted into a weighted health state index by applying scores from “value sets” elicited from general population samples. Following recommendations of the Panel on Cost-effectiveness in Health [55], quality-adjusted life years (QALYs) is used as the measure of effectiveness. QALYs are determined by estimating the utility value for several pain categories ranging from severe pain (KOOS ≤25 on 0–100 scale) to no pain (KOOS ​= ​100), each category will have utility value. The utilities will also depend on the number of comorbidities and obesity class [56].

We discount costs and effectiveness valuations 3 ​% per year. The cost-effectiveness analysis covers the trial duration and the remaining lifetime. The lifetime analysis will involve projecting outcomes and costs beyond the course of the trial using the validated OAPol model [51,57,58].

### 3.12 Statistical considerations

#### 3.12.1 Data management

The Data Coordinating Center has primary responsibility for randomization, quality control, and analyses of data generated by the clinical centers. Most participant data are collected with direct entry into the study electronic data capture system. In some instances (e.g., dietary screener) data are collected electronically at each center and transferred to the database. The web-based management system (PatientIQ) assures integrity and validity. Only specific Data Coordinating Center and Clinical Coordinating Center personnel have access to the data, with restrictions by site and study role. Dynamic reports and periodic statistical analyses monitor quality. A participant inventory system tracks recruitment, retention, adherence, and missing data from entry through exit, close-out, and lock-down of data.

#### 3.12.2 Statistical analysis

##### 3.12.2.1 Primary outcome

The primary hypothesis of reduced long-term risk of MRI-based OA is tested using a modified Poisson regression model [59] where OA presence (tibiofemoral and/or patellofemoral OA) at 48 months is the binary outcome and treatment assignment as the main predictor, adjusted for randomization strata, clinical center, and baseline BMI [59]. The modified Poisson regression model focuses on relative risk. Unlike logistic regression, which may produce invalid relative risk estimates, the modified Poisson procedure permits robust variance estimation and allows unbiased estimation of both model-adjusted relative and absolute risk, per CONSORT guidelines [60]. Currently, the modified Poisson regression model does not handle clustered data, although it is possible that participants within a clinical center are more similar than participants across centers. A sensitivity analysis will use logistic regression (OA or no OA as binary outcome) with mixed effects (with random effect for treating center as cluster) to the data. The primary treatment effect is tested using a 2-tailed Likelihood Ratio test assuming a Type I error rate of 0.05. Participants are analyzed according to their randomized group in accordance with intent-to-treat principles.

##### 3.12.2.2 Secondary outcomes

Secondary outcomes are analyzed using repeated measures mixed linear models. Measures collected annually (months 0, 12, 24, 36, and 48) include KOOS pain, 6-min walk distance, SF-36 physical and mental subscales, weight loss self-efficacy, and exercise self-efficacy. Knee compressive force (baseline N ​= ​660) and IL-6 (baseline N ​= ​910) are measured at baseline and 48 months at two and three centers, respectively. The treatment effect for log-adjusted IL-6 is estimated using analysis of covariance adjusted for baseline log IL-6, baseline BMI, and clinical center and tested at a two-tailed 0.05 Type I error rate. Each repeatedly measured outcome treatment effect is modeled and tested using a two-tailed significance level of 0.05 using contrast statements from a repeated measures mixed linear model with time, randomization arm (D ​+ ​E vs C), and the group ​× ​time interaction, which adjusts the means at each time point for potential missing data bias. Intervention-effect estimates are adjusted further for baseline values of each outcome, baseline BMI, and center; the analysis will match design, so the variance estimate will not be biased. Outcomes at 48 months are of primary concern, with secondary consideration of other time points. An unstructured covariance matrix is used to account for the correlation between repeated outcomes. In the unlikely event the model does not converge, a first-order autoregressive (AR [1]) covariance structure is fit instead [61]. Maximum-likelihood techniques estimate parameters. Preliminary analyses check the shape of the distributions and variances between groups and as a function of the covariates of the prespecified models. Regression diagnostics and residual plots help to find appropriate transformations.

Sensitivity analyses will account for missing data in accordance with the recommendations of the National Research Council [62]. Models will include variables from previous visits determined to predict loss to satisfy Little and Rubin's [63] conditions for data considered missing at random (MAR). If “informative censoring” occurs, comparisons using subjects with complete data, multiple imputation, or explicit modeling of the censoring mechanism are used [63].

### 3.13 Sample size and power calculations

Sample size calculations used data from the Osteoarthritis Initiative (OAI) Biomarkers of Early Arthritis of the Knee (BEAK) dataset (Kwoh, K., PI). The BEAK study examined MRI parameters including semi-quantitative MOAKS scores (Table 4) among a subgroup of radiographic OA-free (KL ​= ​0, 1) and MRI OA-free participants in the OAI cohort [64]. Participants were randomly selected from 2987 OAI participants who had at least one knee at risk for radiographic OA at baseline. MRI-based OA (MRI-OA) incidence was determined using the definition of Hunter et al. [19] to identify participants without MRI-OA at baseline and to estimate progression to MRI-OA using Poisson regression. Among all subgroups (age x sex x BMI), women aged ≥50 ​yrs with a BMI ≥30 ​kg/m^2^ were at highest risk for MRI-OA. This subgroup of women progressed to MRI-OA at a rate of 7.6 ​% (95 ​% CI: 4.2 to 13.8) annually while all potential subgroups of men averaged ≤5 ​% annually. Hence, women aged ≥50 ​yrs with a BMI ≥30 were most likely to benefit from a prevention study. Cumulatively, 27.1 ​% of the control knees in TOPS are predicted to progress to MRI-based structural knee OA by 48-month follow-up. A post hoc analysis of the PROOF data showed that participants that achieved a ≥5 ​% weight loss during the first year had a 67 ​% risk reduction in symptomatic and radiographic OA at 6-year follow-up compared to those with minimal weight loss or gain [65]. Using these estimates, a 30 ​% reduction in OA risk using a 48-month dietary weight loss and exercise intervention compared to control with 80 ​% retention (5.4 ​% attrition/year) requires 615 participants per arm (492/arm evaluable), or 1230 total, to achieve 85 ​% power based on a 2-tailed chi-squared test at the 0.05 level.

For secondary outcomes, this sample size provides the ability to detect a small to moderate treatment effect size of 0.191 (Cohen's d) at 85 ​% power (N ​= ​615, with 492 (80 ​%) evaluable at 48 months) using a 2-tailed *t*-test with a Type I error rate of 0.05. This degree of retention results in 85 ​% power to detect the group differences observed in Table 6. Significant treatment effect estimates are compared to clinically meaningful thresholds for each outcome when available to ensure both statistical and clinical significance.

**Table 6:** Based on N ​= ​615/arm, 2-tailed alpha ​= ​0.05, 80 ​% retention at 48 months.

Variable	SD of Change from Baseline	Detectable treatment effect for 85 ​% power
KOOS Total Score^a^	17.0	3.25
KOOS Pain	18.5	3.54
6-Minute Walk Distance (m)	75	14.3
SF-36: Physical Subscale^b^	8.7	1.7
TF Compressive Force (Newtons)^c^ (N ​= ​269/arm)	290	84.0
Log-IL6 (N ​= ​492/arm)^d^	0.6	0.13
Weight Loss Self Efficacy	15	2.9
Exercise Self Efficacy	28	5.3

a KOOS: The Knee Injury and Osteoarthritis Outcome Score.

b SF: Short Form Survey.

c TF: Tibiofemoral.

d IL-6: Interleukin 6.

## 4 Discussion

Implementing a randomized clinical trial designed to prevent incident knee OA presents numerous challenges. OA disease status and severity are defined structurally via x-ray [36] or MRI [19] and clinically using a combination of symptoms reported by the patient and derived from a physical exam [66]. The primary outcome measure for TOPS is structural knee OA using MRI, due to its superior sensitivity and granularity compared to radiographic assessments. In addition, the use of radiographic assessments as the primary outcome measure to determine the presence or absence of incident knee OA would have increased the sample size by 478 participants (39 ​%), requiring additional clinical centers and greater costs to test the hypothesis effectively.

Losing weight and preventing weight regain are difficult [67]. Biological changes fight attempts to maintain weight loss; the body acts in starvation mode increasing feelings of hunger, satiety is suppressed, metabolic rate slows, all in an attempt to defend higher body weights [68]. Psychosocial obstacles include decreased self-efficacy, increased chronic perceived psychosocial stress, and using food for comfort; environmental obstacles include large food portions and food availability [69]. Our previous work provides encouragement that most of the weight-loss attained by the TOPS diet and exercise group can be retained long-term with implementation of the weight-loss maintenance program. In a subsample of the IDEA cohort (N ​= ​94), the diet-only group retained 5.8 ​kg (65 ​%) of an 8.9 ​kg weight loss 3.5 years following completion of the 18-month diet intervention [70]. Maintenance of this clinically important weight-loss occurred without any post-intervention interaction with the study staff.

A limitation of this study is the exclusion of male participants. This was based on a preponderance of data that places females at nearly twice the risk for incident knee OA than males [18]. Furthermore, the estimated lifetime risk of knee OA for people with obesity is 16 ​% for males and 24 ​% for females [71]. There are also sex differences in OA symptoms - worse in females [72,73], cartilage volume - females have less cartilage and more cartilage volume loss [74], knee laxity - females have greater knee laxity [75], hormonal influences – decreased estrogen after menopause [76,77], and gait differences – greater knee kinetics linked to more tibiofemoral compartment loading [78]. Taken together, these differences place females at greater risk for the development of knee OA. Additionally, the inclusion of males would have increased the sample size by 32 ​%, required additional clinical centers, increased costs substantially, and added to the logistical complexities managed by coordinating center staff.

The PROOF clinical trial was an initial attempt at implementing a trial designed to prevent incident knee OA. The rate of incident knee OA was not different between the diet and exercise (N ​= ​101) and control (N ​= ​102) groups after a 2.5-year intervention period. A low adherence rate (28 ​%), however, resulted in only 15 ​% of the diet and exercise group achieving a 5 ​% weight loss [38]. A post-hoc analysis revealed that participants who did achieve the 5 ​% weight loss goal by the end of the first year reduced the risk of symptomatic and radiographic knee OA by 67 ​% at 6-year follow-up compared to those with minimal weight loss or weight gain in year one [65]. The use of a social cognitive conceptual framework for implementing problem-solving strategies and structuring a positive environment designed to improve adherence and retention [30], a larger sample size, a 4-year intervention period with a weight-loss maintenance program built-in, and a 10 ​% weight-loss goal are design improvements that are being implemented in the current trial.

The importance of OA disease prevention is evident because there are presently no effective disease-modifying interventions, there are substantial safety concerns associated with many pain medications, and there is no cure. TOPS will evaluate a critically needed primary prevention intervention on females at risk for the development of knee OA by implementing a program of dietary weight loss, exercise, and weight-loss maintenance designed for long-term sustainability to maximize health benefits at a reasonable cost.

## Author contribution

Dr. Messier and Dr. Beavers had full access to all of the data included in this article and take full responsibility for the integrity of the data and accuracy of the information.

Conception and design: Messier, Hunter, Mihalko, Callahan, Losina, Katz, Loeser, Newman, DeVita, Spindler, Runhaar.

Collection and assembly of data: Beavers, Messier.

Analysis and interpretation of data: Beavers, Messier, Hunter, Mihalko, Callahan, Losina, DeVita, Spindler, Runhaar, Ip.

Drafting of article: Messier.

Critical revision of the article for important intellectual content: All authors.

Obtaining of funding: Messier, Callahan, Losina, Mihalko, Guermazi, Miller, Katz, Loeser, Pietrosimone, Soto, Cook, Newman, Hill, Love, DeVita, Spindler, Runhaar, Beavers, Hunter.

## Funding

Research reported in this publication is supported by the National Institute of Arthritis and Musculoskeletal Skin Diseases under Award Number 1 U01 AR082121-01, Arthritis Foundation, National Center for Complementary and Integrative Health, Office of Disease Prevention, Office of Research on Women's Health, Office of Behavioral and Social Science Research, Centers for Disease Control and Prevention, University of Missouri, Wake Forest University, and Rapid Nutrition PLC.

## Conflict of interest

Dr. Katz and Dr. Losina received research grant funding from Samumed and Flexion Therapeutics. Elena Losina has also received research funding from Pfizer. Dr. Hunter received research funding from Pfizer, Novartis, Lilly, Merck Serono, TLC Biopharmaceuticals and Kolon. Dr. Guermazi is a consultant for Pfizer, Novartis, TissueGene, Coval, Medipost, ICM and TrialSpark, and is a shareholder in Boston Imaging Core Lab. Kurt Spindler is a consultant for the National Football League and Novopedics, Inc. Dr.Cook receives research support from AO Trauma, Arthrex, Inc., Collagen Matrix Inc., DePuy, A Johnson & Johnson Company, Musculoskeletal Transplant Foundation, Orthopaedic Trauma Association, Purina, Regenosine, SITES Medical, Thieme, and Trupanion.
